# Identifying intersectional feminist principles in the One Health framework

**DOI:** 10.1016/j.onehlt.2022.100404

**Published:** 2022-05-31

**Authors:** Emily Hardy, Claire J. Standley

**Affiliations:** a Walsh School of Foreign Service, Georgetown University, 3700 O ST NW, Washington DC 20057, United States of America; b Center for Global Health Science and Security, Georgetown University, 3900 Reservoir Rd NW, Washington DC 20007, United States of America

**Keywords:** Feminism, One health, Queer studies, Women and gender studies, Interdisciplinarity, Intersectionality

## Abstract

• The relationship between Feminist theory and One Health is understudied, but is complementary and even symbiotic.
• One Health successfully *Queers* the Enlightenment health binary that artificially divorces human and non-human health.
• One Health investigates the points of interconnection and overlap much like the Feminist concept of intersectionality.

## 1 Introduction

One Health is not a new concept in the study of global health, yet its connection to feminist theory has often been ignored. Since the One Health framework encourages the decomposition of artificial binaries and emphasizes the overlap of different systems, it is deeply connected to intersectional feminist thought, regardless of whether such integration was intentional or explicit. In this piece, we elucidate the interconnected and even symbiotic relationship between feminism and One Health, with the intent of helping both health and gender researchers gain a deeper understanding of more broadly integrated approaches to their work.

## 2 Definitions

The term One Health came into formal academic usage following the 2003–2004 outbreak of SARS, a health crisis that prompted the creation of the 12 Manhattan Principles and the development of a “One World, One Health” framework. [1] As a result, institutions from the Global North tend to assume that One Health is a relatively modern concept. It's worth noting; however, that the approach has been embodied in land usage by Indigenous communities for centuries. [2] Explicitly tracing the lineage of knowledge and crediting marginalized communities with idea ownership is fundamental to intersectional feminist thought. As such, this piece is indebted to and acknowledges the contributions of Indigenous communities to One Health.

Fundamentally, the philosophy of One Health emphasizes collaboration across three primary fields of study (animal, human, and environmental) with the hopes of producing the most effective health outcomes for all beings and the broader ecosystem. It is centered on the belief that human health does not exist in isolation, and therefore, should be analyzed as one element of a larger matrix. The One Health approach encourages researchers, policy makers, ecologists, biodiversity experts, veterinarians, and medical professionals to consider health as a complicated and multifaceted variable composed not exclusively of humans but of all the other elements in our ecosystems.

Additionally, this paper has chosen to view Queer theory as an outgrowth and component of intersectional feminist thought rather than as a separate body of scholarship. Resisting the belief that feminism must only study the experiences of (cis-)women, we support the definition that Queer and gender studies seeks to “oppose the structures and institutions that reproduce the conditions and concepts of normativity. [Q]ueer as a stance…decentralizes whatever becomes central in order to speak from the margins of a changing set of normativities.” [9] The re-centering of marginal groups is central to Kimberlee Crenshaw's legal and feminist concept of intersectionality, which considers how the needs of groups differ at points of intersection with other identities. [4] Crenshaw writes about intersectionality in the context of legal discrimination against Black women; however, her principles have been applied to many overlapping systems of power. Traditional feminism, critiqued for its prioritization of privileged womens' experiences, has sought to rectify its exclusionary practices through the incorporation of Crenshaw's theory on intersectionality. Intersectional feminist thought disrupts the belief that feminism can consider identities in isolation. As such, viewing Queer and feminist thought as distinct intellectual bodies neglects the teachings of Crenshaw and feminism's role in studying the oppression of all marginalized gender identities, which is critically important in global health, given the under-representation of queer experience. [5]

## 3 The overlap between intersectional feminist thought and One Health

Although feminist values were never made explicit in the Manhattan Principles (nor in the updated Berlin Principles, unfortunately), the lens of intersectional feminist thought is implicitly embedded in the One Health framework. [6] This recognition matters to One Health because its corrective intention (ie. addressing the isolation of health variables) cannot be separated from larger problems of the medical establishment, namely its history of anti-feminist practices. In attempting to build a more integrated approach to care, One Health must consider how identities like gender impact health outcomes, which itself builds on past and current scholarship centering feminism in health. [11,12] Situating One Health within intersectional feminism demonstrates the conceptual overlap between gender and health; ultimately encouraging future collaboration across the two disciplines.

### 3.1 Queering health through the One Health model

The need to explicitly define and label health was born out of the Enlightenment tradition that sought to impose limits on the natural environment. [7] Enlightenment thinkers were deeply invested in the use of organization and division as a tool for scientific advancement, often imposing division onto flora, fauna, and eventually humans. Although societies have historically engaged in social organization, compelling evidence suggests that the natural impulse towards cognitive stratification was solidified during the expansion of Western colonialism, often at the expense of marginalized communities. [8] For example, many “modern racial classification can be understood as an ‘overextension’ of biological classification more generally” that was popularized during the enlightenment era. [8]

The Manhattan Principles were developed to reconcile the unnecessary divisions erected between health sectors. Seeing health as strictly human or non-human ingrained dangerous yes/no, black/white, good/bad binary thinking into health studies. However, the prioritization of separation has manifested in hierarchies that are still maintained by the academic community. Normative assessments of scientific merit have produced stratification within health, often privileging research that centers humans over inquiries focussed on the environment and wildlife. This favoritism is subsequently reflected in the inequitable distribution of resources across health disciplines and is incompatible with the true realization of One Health principles. Intersectional and Queer theory intentionally “speak[s] from the margins,” redefining power relationships and undermining hierarchies, such as merit rankings implicitly observed across health disciplines. [9]

At the most fundamental level, the One Health approach rejects false binary and exclusive thinking that considers variables in isolation. This framework can be likened to the attempts made by Queer scholarship to blur conventional binaries of sexuality and gender, an act often referred to as *Queering.* Although the word Queer is commonly used as an umbrella term to describe many identities housed under the LGBTQIA2+ acronym, it has three functions in speech. For example, “Queer can be used as a noun,—‘I am Queer.’—as an adjective,—‘She is a Queer woman.’—and as a verb,—‘to Queer the mind.’” [3]. The act of blurring and challenging conventional binaries uses Queer as a verb, hence *queering*.

Correcting the artificial separation of health categories advances human well-being and provides the foundation for a more integrated approach to prevention of disease and long-term care. In the realm of health studies, humans are, on a fundamental level, animals. As such, it is illogical to divorce the human species from its ecological and evolutionary context. Enforcing the binary of human health versus non-human health is a fundamental misrepresentation of science.

Neat categorization of human, environment, and animal, (or from a feminist perspective, “man” versus “woman”), is appealing because the distillation simplifies complicated topics into defined boxes, instead of forcing us to reckon with the spectrum of critical interactions occurring across categories. However, understanding variables (like health, gender or sexuality) as rigid, inflexible, and static fails to provide the necessary gradience inherent in the real world experience. From a traditional gendered perspective, the medical invention of sex, a term developed through the imposition of a male versus female binary genitalia construct, continues to misrepresent diverse intersex identities. Similarly, through the human-environment-animal simplification processes, the most nuanced and significant details of health considerations are artificially separated, rendering them understudied and ignored. Yes, it is daunting to embrace interconnection and instability in fields that privilege binaries; however, this simplistic framework fails health just as it fails gender. Employing a One Health lens successfully *queers* the health binaries imposed by Western biomedicine onto human, animal, and environmental health. As such, the two schools of thought should find themselves natural allies when discussing improvements to health access and equity.

### 3.2 Identifying intersectional and interdisciplinary feminist approaches in One Health

The second notable integration of feminist principles into One Health is its implicit reliance on intersectionality and interdisciplinary frameworks. One Health prioritizes the interconnectedness of different variables and the explicit intention of locating solutions that operate at the intersection of human, environmental, and animal health. The emphasis on interconnection can be likened to Kimberlee Crenshaw's legal and feminist concept of intersectionality. Although intersectionality may seem disconnected from health, a central piece of Crenshaw's thesis– that oppression and solutions look different at places of intersection–is relevant to One Health concerns. In the context of scientific research, solutions to health challenges look different when variables are considered holistically and at places of intersection. [5] To liken the concept of intersectionality to health studies is not to appropriate Crenshaw's initial invention or the terms usage as a legal concept and feminist tool. Rather it is to emphasize how the integral values of intersectionality are both relevant and already present in the One Health approach.

Understanding and embracing intersection produces better health outcomes. For example, studying warming temperature as a result of climate change may reveal increased habitat suitability for mosquito populations– a potential cause of increased yellow fever infections in a particular area, which in turn might be impacted by changing behaviors in non-human primates who are also vulnerable to yellow fever virus. Considering how different factors are integrated and overlapping is the epitome of an interconnected (and intersectional) approach. Finding solutions at the point of intersection, is an approach hailed by the Combahee River Collective who envisioned a world “in which Black women, and thus all of humanity, were freed from systems of oppression.” [10] Simply put, solutions that work for the most marginalized identities (at the points of intersection) create policies that work for all other identities because for the most oppressed to be liberated, systems of oppression must be fully dismantled. In the context of One Health, centering solutions that operate at points of intersection would necessitate policies that consider how animals and the environment interact with human health– ultimately yielding the best outcome. There is an implicit conceptual overlap between Crenshaw's term and the One Health model because when policies and practices consider how all relevant health variables fit together, it becomes possible to create meaningful change.

## 4 Conclusion

Principally, One Health is more than a human-centric Global Health philosophy disguised as a cross-disciplinary approach. It is a direct response to the harms inflicted by the siloing of health categories. Identifying the role of interconnection and interdependence on outcomes provides researchers with the tools needed to advocate for all actors in shared ecosystems.

At the most fundamental level, One Health challenges reductionist views of health just as feminist thought challenges binary notions of gender, sex, and sexuality. However, queering One Health does not necessarily require the abandonment of all labels. Many Queer people simultaneously recognize the value of having/using labels *and* rejecting the rigidity of binary frameworks that negate fluidity. Distinct categories (like human, environment, or animal) are not intrinsically bad; however, they become dangerous when considered in isolation or as inflexible and *natural* rather than as products of culture. Instead, identifying nuance through the study of intersection points (ie. where gender meets sexuality or where animals interact with the environment) is far more meaningful than erasing the existence of categorization in its entirety. When held in tandem with Crenshaw's framework, this paper advocates for an approach that both loosens the restrictions of categorization, whilst recognizing the value of distinctions across identities and/or entities.

Whether or not the authors of the Manhattan Principles were cognizant of the overlap, a symbiotic relationship exists between feminism and One Health. Operating with an intersectional feminist lens resists limited and flawed outcomes produced by reductive systems that view health in isolation. Identifying the relationships between the two disciplines may help health scholars recognize the feminist implications of their work, potentially even widening the door for further collaboration across gender and health disciplines. When practicing One Health, researchers must continue to queer health categorizations and consider solutions at points of intersection (both central to intersectional feminist thought) in order to holistically study the *sum* of health's malleable, interconnected parts.

## Declaration of Competing Interest

None.
